# Implementation of the Business Process Modelling Notation (BPMN) in the modelling of anatomic pathology processes

**DOI:** 10.1186/1746-1596-3-S1-S22

**Published:** 2008-07-15

**Authors:** Marcial García Rojo, Elvira Rolón, Luis Calahorra, Felix Óscar García, Rosario Paloma Sánchez, Francisco Ruiz, Nieves Ballester, María Armenteros, Teresa Rodríguez, Rafael Martín Espartero

**Affiliations:** 1 Department of Pathology, Hospital General de Ciudad Real, Calle Tomelloso s/n. 13004 Ciudad Real, Spain; 2 Department of Technology and Information Systems, School of Informatics, Universidad de Castilla-La Mancha, Paseo de la Universidad, 4. 13071 Ciudad Real, Spain; 3 Quality Assurance Unit, Hospital General de Ciudad Real, Calle Tomelloso s/n. 13004 Ciudad Real, Spain

## Abstract

**Background:**

Process orientation is one of the essential elements of quality management systems, including those in use in healthcare. Business processes in hospitals are very complex and variable. BPMN (Business Process Modelling Notation) is a user-oriented language specifically designed for the modelling of business (organizational) processes. Previous experiences of the use of this notation in the processes modelling within the Pathology in Spain or another country are not known. We present our experience in the elaboration of the conceptual models of Pathology processes, as part of a global programmed surgical patient process, using BPMN.

**Methods:**

With the objective of analyzing the use of BPMN notation in real cases, a multidisciplinary work group was created, including software engineers from the Dep. of Technologies and Information Systems from the University of Castilla-La Mancha and health professionals and administrative staff from the Hospital General de Ciudad Real. The work in collaboration was carried out in six phases: informative meetings, intensive training, process selection, definition of the work method, process describing by hospital experts, and process modelling.

**Results:**

The modelling of the processes of Anatomic Pathology is presented using BPMN. The presented subprocesses are those corresponding to the surgical pathology examination of the samples coming from operating theatre, including the planning and realization of frozen studies.

**Conclusion:**

The modelling of Anatomic Pathology subprocesses has allowed the creation of an understandable graphical model, where management and improvements are more easily implemented by health professionals.

## Background

Orientation to processes is one of the essential elements of Quality Management Systems, including those currently in use in healthcare. Design is of special importance throughout the process life-cycle [1]. This phase refers, mainly, to the process modelling, that is to say, to the right representation of its performance and other relevant aspects. BPMN (Business Processes Modelling Notation) is a language specifically designed for the modelling of business (organizational) processes [2]. It has met with general acceptance in business and institutional circles thanks to the fact that it has been devised to be used and understood by any kinds of roles (directors, experts in quality, business analysts, system analysts, engineers, etc.), with no need of special technical knowledge. BPMN provides a graphic notation expressing all the aspects of the processes by a single type of diagram [3].

Previous experiences of the use of this notation in the processes modelling within the health sector in Spain or another country are not known. Given the diversity and complexity of the processes in a hospital, the implementation of BPMN may be very useful. We present our experience in modelling Anatomic Pathology processes within the programmed surgical patient (PSP) process in our hospital.

## Methods

A multidisciplinary work group was created. It was composed of software engineers from the Alarcos Research Group of the University of Castilla-La Mancha (UCLM) and health professionals and administrative staff from the Hospital General de Ciudad Real (HGCR) which is a part of the Health Care Services (SESCAM) of the Spanish region of Castilla-La Mancha.

The applied research method was Action-Research (A-R). A-R is a collaborative research method aimed at joining theory and practice between researchers and practitioners by means of a process of a cyclical nature.

The work in collaboration was carried out in six phases (informative meetings, intensive training of health professional sin BPMN, selection of possible processes, definition of the work method (including subgroups creation), defining the selected processes, and development of the process model.

The hospital experts in the "programmed surgical patient" process developed a record that included: mission of the process, limits, clients, responsible persons, participants and its duties, glossary of terms, suppliers for the process, description of activities and documents and register. The experts in BPMN of the University made the modelling of the process and its subprocesses through a reiterative and incremental method: They added progressively details, until reaching the level requested by the Hospital. The approval of the results was giving through joint revisions of each iteration until the final version was obtained.

## Results

### Programmed surgical patient process

**Figure 1:**
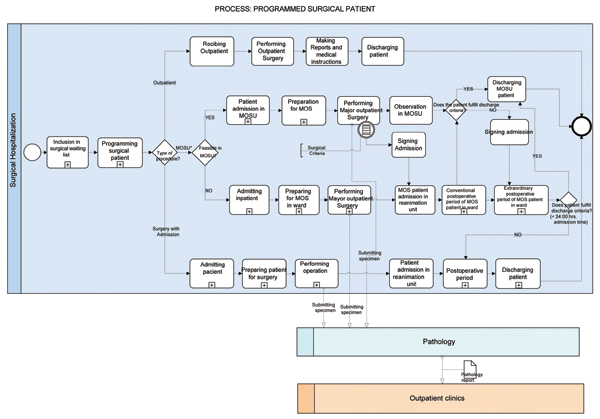
**Programmed surgical patient process model**. MOSU: Major Outpatient Surgery Unit.

The process model presented in figure 1 shows at a high level abstraction the PSP process and the activities that are carried out when a patient is admitted into the hospital for the accomplishment of the surgical treatment of a disease that has been clinically diagnosed previously.

### Surgical pathology processes

**Figure 2:**
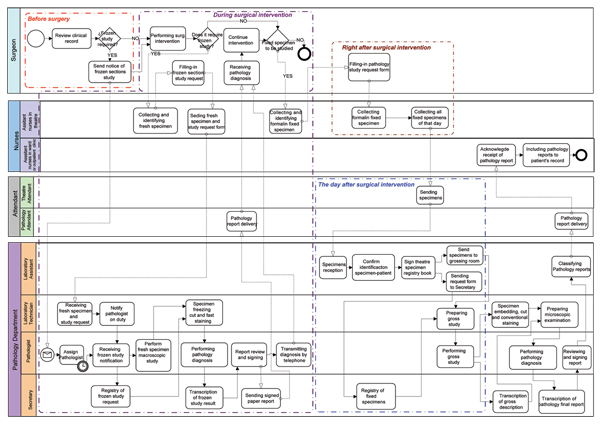
Surgical Pathology process model.

The modelling of the processes of Anatomic Pathology is presented through the notation BPMN (fig. 2). The presented subprocesses are those corresponding to the surgical pathology examination of specimens coming from operating theatre, including the frozen studies and the complete internal circuit in the Pathology department that results in the pathology report and its submission to the corresponding clinical department.

## Discussion

In this work, we used the BPMN for pathology processes modelling. We have selected this notation because it is widely accepted and recognized in the enterprise market due to the easiness that it provides for the construction of simple or high level processes [3]. In this case, the model has to be as simple, transparent and understandable as possible for all the stakeholders in the health sector [4].

Modelling pathology process is especially important nowadays, since the Integrating the healthcare Enterprise (IHE) initiative is defining the first profiles of the technical framework in pathology. Standardization efforts are progressing to provide integration in healthcare information systems, such as CEN TC 251 (pr EN13606), HL7, DICOM, etc. However, integration in Pathology Information Systems has not yet been achieved [5]. The IHE initiative is both a process and a forum for encouraging integration efforts. It defines a technical framework for the implementation of established messaging standards to achieve specific clinical goals. It includes a rigorous testing process for the implementation of this framework, organizes educational sessions, exhibits at major meetings of medical professionals to demonstrate the benefits of this framework and encourage its adoption by industry and users. This work can become easier if health institutions know well and improve their processes by sing BMPN modelling.

## Conclusion

Creating a multidisciplinary working group has been an efficient method to analyze the use of BPMN notation in real cases in healthcare.

The modelling of the programmed surgical patient process and its subprocesses has allowed to us to prepare an understandable model for the involved health professionals and make the communication of processes easier. Additionally, modelling allows early detection and correction of errors. This work is an essential previous step for further analysis and improvements in healthcare processes, including the adoption of information technology standards.
